# PDBe CCDUtils: an RDKit-based toolkit for handling and analysing small molecules in the Protein Data Bank

**DOI:** 10.1186/s13321-023-00786-w

**Published:** 2023-12-02

**Authors:** Ibrahim Roshan Kunnakkattu, Preeti Choudhary, Lukas Pravda, Nurul Nadzirin, Oliver S. Smart, Qi Yuan, Stephen Anyango, Sreenath Nair, Mihaly Varadi, Sameer Velankar

**Affiliations:** Protein Data Bank in Europe, European Molecular Biology Laboratory, European Bioinformatics Institute (EMBL-EBI), Wellcome Genome Campus, Hinxton, Cambridge, CB10 1SD UK

**Keywords:** PDB, RDKit, Ligand, Protein structure, Python, CCD, Covalently Linked Components, CLC, PRD, BIRD, PDBx/mmCIF

## Abstract

While the Protein Data Bank (PDB) contains a wealth of structural information on ligands bound to macromolecules, their analysis can be challenging due to the large amount and diversity of data. Here, we present PDBe CCDUtils, a versatile toolkit for processing and analysing small molecules from the PDB in PDBx/mmCIF format. PDBe CCDUtils provides streamlined access to all the metadata for small molecules in the PDB and offers a set of convenient methods to compute various properties using RDKit, such as 2D depictions, 3D conformers, physicochemical properties, scaffolds, common fragments, and cross-references to small molecule databases using UniChem. The toolkit also provides methods for identifying all the covalently attached chemical components in a macromolecular structure and calculating similarity among small molecules. By providing a broad range of functionality, PDBe CCDUtils caters to the needs of researchers in cheminformatics, structural biology, bioinformatics and computational chemistry.

**Graphical Abstract:**

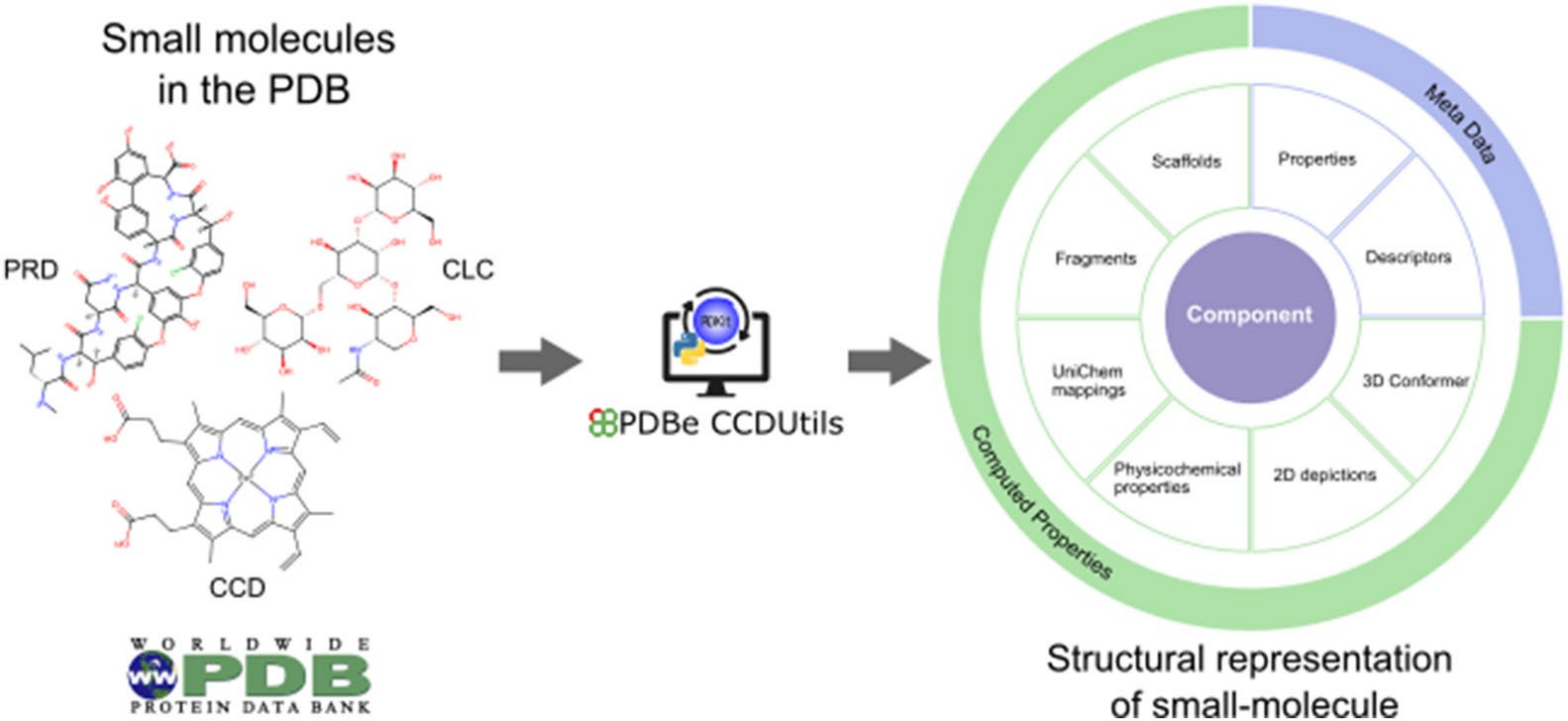

## Introduction

The Protein Data Bank (PDB) [1], managed by the worldwide PDB (wwPDB) consortium [2], serves as the single global repository for information on 3D structures of proteins, nucleic acids, and complex assemblies. With over 200,000 entries as of July 2023, about 75% of these structures contain at least one small molecule bound to a protein or nucleic acid. Some small molecules are present due to experimental necessities, such as aiding crystallisation [3–5] or enabling cryoprotection [6–8], while others play biologically significant roles acting as cofactors, metabolites, or drugs [9–12]. Information on ligand conformations and their interaction with macromolecular partners is crucial for deciphering their role in biological function and mechanisms.

To maintain standardised and accurate data on these small molecules, the wwPDB maintains Chemical Component Dictionary (CCD). This comprehensive reference resource contains data for all unique chemical components, including individual protein and nucleic acid residues and small molecules found in PDB entries [13]. CCD provides chemical description, composition, connectivity, and idealised coordinates for every unique chemical component.

During the wwPDB annotation process, each deposited structure undergoes careful processing to identify individual chemical entities, which are then compared against the already existing CCD [14]. For any small molecules that are new to the PDB, a dictionary description is created from the first PDB entry with the most complete structure of the small molecule [14]. Despite playing a significant role as a reference dictionary, the CCD description has certain limitations due to the nature of deposited data and wwPDB annotation practices. The wwPDB annotation guidelines suggest the CCD definition represents a neutral form for every compound [15]. In some cases, ligands may be refined and deposited as a set of covalently linked components, where each component represents a distinct CCD component. In other cases, ligands with peptide linkages may be split into the respective CCD components during annotation [14]. As a result of these structure determination practices or wwPDB annotation policies, ligands are fragmented into smaller components in almost 6% of PDB entries, limiting a straightforward interpretation of the complete ligand and its function. Due to such fragmentation practices being applied to large ligands, some CCDs may not represent a chemically reasonable ligand on their own, leading to inaccuracies in representing the actual chemical structures of small molecules. These limitations can lead to incomplete or incorrect interpretation of ligand interactions and hinder mechanistic insights into protein function. To mitigate these issues, the wwPDB developed a new reference dictionary named Biologically Interesting molecule Reference Dictionary (BIRD) [16] comprised of chemical, structural and functional information of Peptide-like molecules Reference Dictionary (PRD) entries, specifically addressing the fragmentation issue for peptide-like inhibitors and antibiotic ligands [16]. Nevertheless, other complex ligands in the PDB continue to be fragmented, preventing easy interpretation of the complete chemical structures and their interactions with macromolecules. Additionally, both CCD and PRD contain minimal data, lacking molecular properties and cross-references to relevant small molecule databases, making comprehensive analyses or integration of relevant information an arduous task. Another persistent challenge is a useful and accurate 2D depiction of CCDs to easily visualise the chemical structures and discern ligand characteristics.

Furthermore, advances in structure determination techniques are resulting in an increasing number of structures for large macromolecular machines [17, 18]. The increasing number of depositions is also accelerating the number of new small molecules in the PDB archive and very shortly the traditional three-letter code will be exhausted, leading to a transition to five-letter CCD identifiers [19]. These developments will result in PDBx/mmCIF becoming the only source of information as both large structures or those that have five-letter CCD identifiers cannot be represented in the legacy PDB format. To help smooth the transition to PDBx/mmCIF and the various challenges highlighted earlier, we have developed a versatile and user-friendly Python package, the PDBe CCDUtils, for handling and analysing small molecules in the PDB.

The core functionality of PDBe CCDUtils is based on RDKit, an open-source cheminformatics toolkit widely used in the scientific community for handling molecular data [20]. RDKit provides many features, including molecular structure manipulation, molecular descriptor calculation, and various cheminformatics algorithms. Building upon RDKit's capabilities, PDBe CCDUtils extends its functionality specifically in the context of PDB data, allowing researchers to overcome the limitations faced with CCD and PRD. By utilising PDBe CCDUtils, researchers in cheminformatics, structural biology, bioinformatics, and computational chemistry can obtain an accurate representation of the small molecule and efficiently analyse the data in the PDB.

### Implementation

PDBe CCDUtils offers a comprehensive suite of functionalities that enhance the handling and analysis of small molecules in the PDB. By serving as a wrapper around essential RDKit functionality, PDBe CCDUtils seamlessly integrates the capabilities of RDKit with PDBx/mmCIF files, empowering users to access a wide array of cheminformatics tools and molecular manipulation techniques for small molecules in the PDB with ease and efficiency. The toolkit facilitates the reading and writing of small molecule reference PDBx/mmCIF files, automatically instantiating RDKit objects representing small molecules and their attributes, streamlining the process of accessing and analysing PDB data for further research.

PDBe CCDUtils also includes vital functionality to validate the chemical sanity of molecules, ensuring accurate representation and adherence to chemical rules, thereby preventing inaccuracies in ligand interactions analysis. The challenge of the 2D depiction of molecules is addressed by the toolkit, offering the option to generate the best 2D depiction using templates or connectivity. This ensures visually meaningful representations of chemical structures, enabling researchers to comprehend ligand characteristics accurately.

To overcome the fragmentation issue caused by multicomponent ligands being represented as separate CCD components in the PDB entries, PDBe CCDUtils introduces Covalently Linked Components (CLCs). By defining CLC molecules that encompass the entire set of individual CCD components which are covalently bonded together, PDBe CCDUtils provides a precise and chemically complete representation of these multicomponent ligands present in the PDB. PDBe CCDUtils also includes functionalities to find scaffolds and search common fragments/sub-structures against a fragment library, enabling researchers to explore structural similarities and analyse essential pharmacophoric elements in small molecules within the PDB. The implementation details of all these core functionalities are discussed in detail below.

### Parsing small molecules reference files to access meta-data and RDKit computed properties

**Fig. 1:**
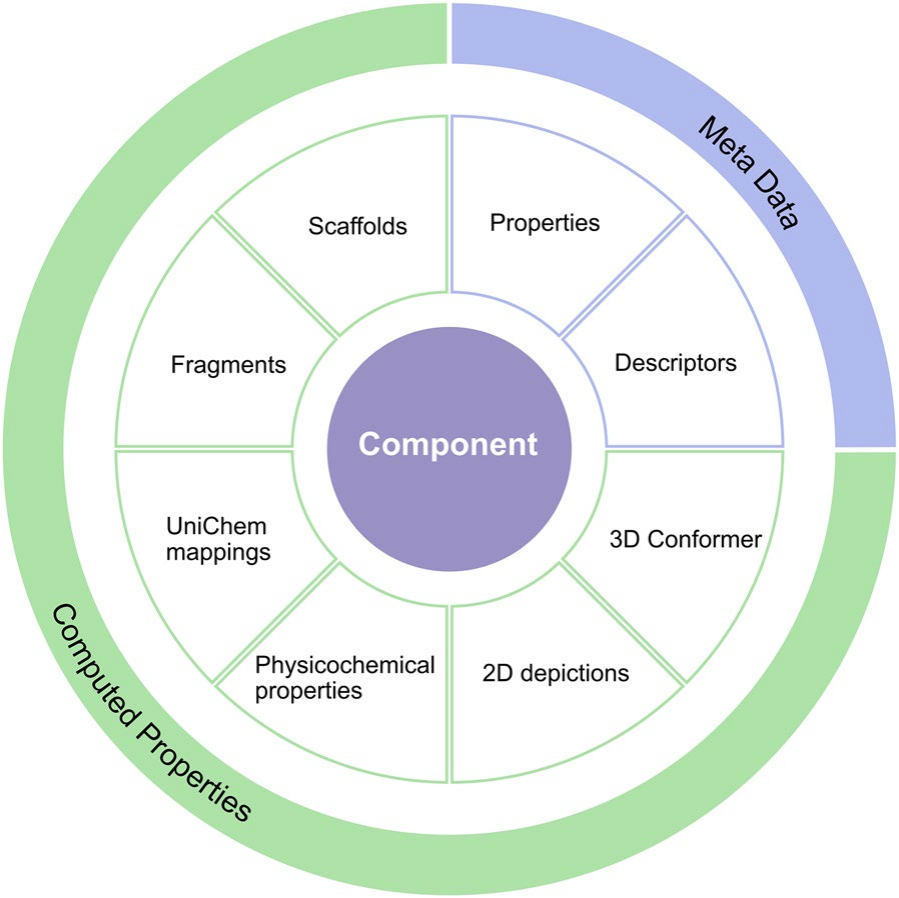
PDBe CCDUtils Component representation. The Component object is the core structural representation of small molecules in PDBe CCDUtils. It provides access to metadata such as descriptions and properties, and computed properties, such as scaffold and fragment information, cross-references from UniChem and physicochemical properties of small molecules

PDBe CCDUtils supports input files in PDBx/mmCIF format, the master format for the PDB archive [21]. This data dictionary format is the basis of wwPDB data deposition, annotation, and archiving of PDB data from all supported experimental methods [21]. The wwPDB FTP area provides access to PDB’s small molecule reference files for CCDs and PRDs in PDBx/mmCIF format [22, 23]. Additionally, the PDBx/mmCIF format files for CLCs are provided in PDBe FTP area [24]. PDBe CCDUtils uses Gemmi, an open-source and efficient PDBx/mmCIF parser [25], for reading and writing these files. After reading the definitions of chemical components from small molecule reference files, they are represented as a Component object in the PDBe CCDUtils library, which is the core structural representation of a chemical component. The Component object is a wrapper around the “rdkit.Chem.rdchem.Mol'' object. The Component object provides easy access to all metadata information encoded in the small molecule reference PDBx/mmCIF file or enriched later through the PDBeChem pipeline, an integral part of PDBe’s weekly release process [26], providing up-to-date and comprehensive small molecule information in the PDB (Fig. 1). The CCDs/PRDs reference files include properties such as id, name, formula, pdbx_release_status, pdbx_modified_data, and descriptors including systematic chemical names and chemical descriptors (SMILES, InChI, InChIKey). The computed properties are generated using the structural data accessible from the ‘mol’ attribute of the Component object. The ‘mol’ attribute is a “rdkit.Chem.rdchem.Mol” object generated from the parsed atom coordinates and their connectivity in the reference file. CCD/PRD components also have ideal coordinates generated by Molecular Network’s Corina [27] or OpenEye’s OMEGA [28] and model coordinates from the PDB entry where the CCD/PRD component is first observed. Correspondingly, an Ideal conformer and Model conformer of type “rdkit.Chem.rdchem.Conformer” are added to the “rdkit.Chem.rdchem.Mol” object to access these coordinates.

After adding atom coordinates and their connectivity to the “rdkit.Chem.rdchem.Mol object”, an augmented sanitisation is carried out using RDKit as described below. Once a Component object is generated for a CCD/PRD, it can be exported to multiple formats. PDBe CCDUtils currently supports exporting to SDF, CIF, PDB, JSON, XYZ, XML and CML formats.

### Augmented data sanitisation process

PDBe CCDUtils also has an augmented data sanitisation process to address representation issues found in certain CCD files, ensuring efficient handling of biological ligands in PDB. For instance, heme (CCD identifier HEM) contains a metalloporphyrin ring with two nitrogens atoms, each with a valency of 4. However, the default ‘rdkit.Chem.Sanitizemol’function in RDKit fails to generate the complete representation of such ligands, with bonds to metal atoms, leading to unusual valency problems and incomplete structures. To overcome this limitation, PDBe CCDUtils implements an iterative sanitisation procedure. Initially, the molecule undergoes sanitisation using the “rdkit.Chem.SanitizeMol “ function, and any unusual valency issues and affected atoms are identified. The augmented procedure then adjusts the bond type between the affected atom and a metal atom to be single while modifying the formal charges accordingly. This iterative procedure is repeated until there are no unusual valency issues reported by RDKit or a maximum of ten times, chosen based on the observation that this results in accurate representations for wwPDB CCD components. By resolving these representation issues, PDBe CCDUtils ensures the generation of accurate and chemically sensible molecules.

### Identifying covalently linked components

**Fig. 2:**
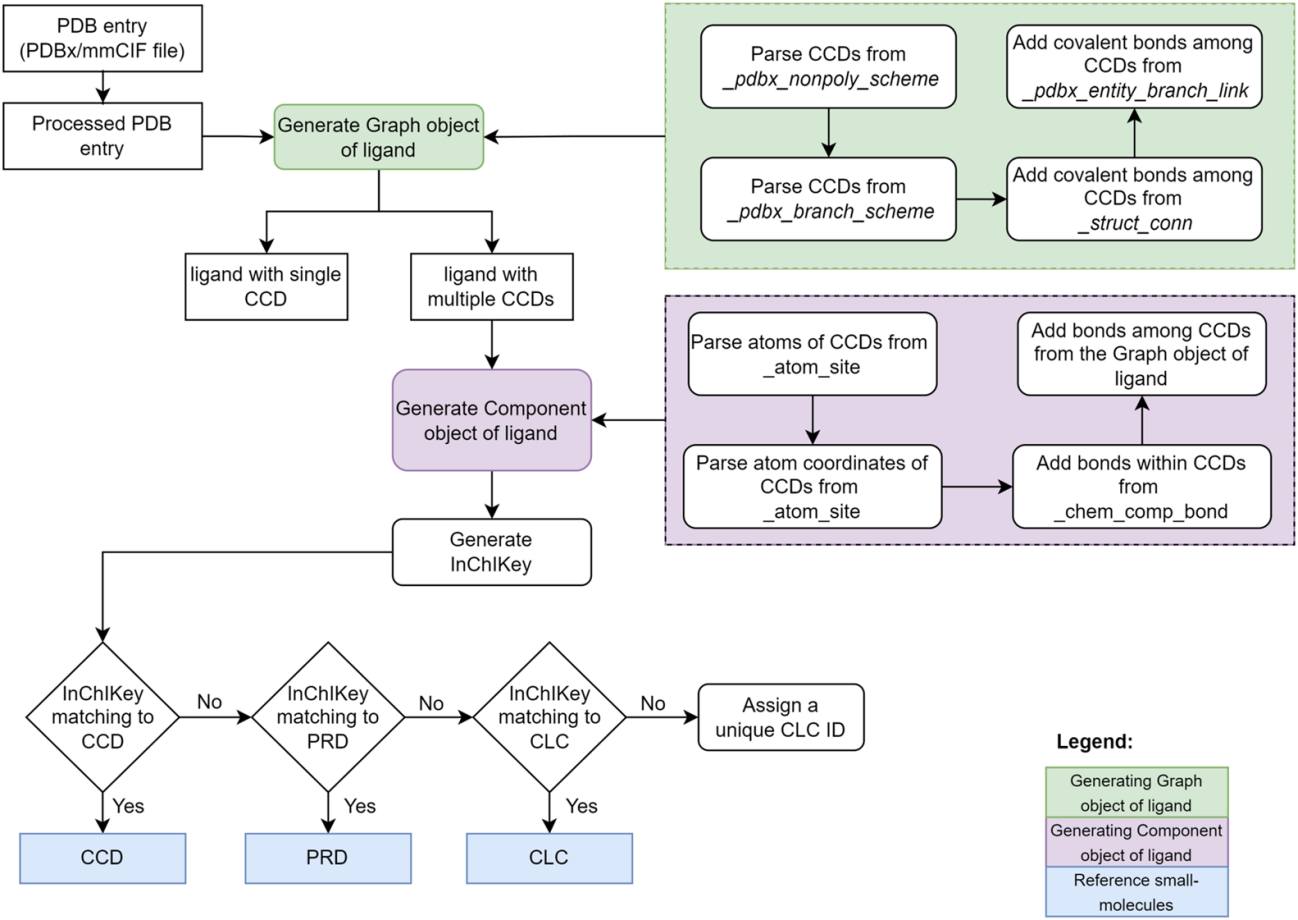
Schematic workflow of identifying unique CLCs. The method identifies Covalently Linked Components by processing a PDB structure file, generating a graph object of ligand with identified CCD components as nodes and covalent bonds as edges, and then converting the graph object into a Component object by parsing atom information and 3D coordinates, and adding bonds within CCD components from the processed PDB entry file and adding bonds among CCD components from the graph object of ligand

Several large ligands in the PDB are split into individual CCD components. For instance, PDB entry 6lq4 is a structure of Acyl-CoA dehydrogenase bound to its substrate Myristoyl-CoA. However, the substrate Myristoyl-CoA is split into Myristoyl (CCD ID: MYR) and CoA (CCD ID: COA). Such splitting of ligands into individual CCD components makes it difficult to correctly identify their biological relevance and their interactions with macromolecules and cross-references to other small molecule databases. The Peptide-like molecules Reference Dictionary (PRD) tackles this fragmentation issue for a subset of peptide-like inhibitors and antibiotic ligands. We have addressed this issue for all the remaining cases by utilising PDBe CCDUtils and defined Covalently Linked Components (CLC) for ligands consisting of multiple covalently linked CCD components, providing a more precise and comprehensive representation of these multicomponent ligands in the PDB (Fig. 2).

Identifying CLCs starts by reading molecular structure data from an individual PDB entry file in PDBx/mmCIF format. The file is then processed to keep only the first model if there are multiple models, then only the atoms with maximum occupancy in case of alternate conformations are considered. Next, a graph object of ligand [29] is generated using CCDs identified from the “_pdbx_nonpoly_scheme” and the “_pdbx_branch_scheme” data categories as nodes and adding covalent bonds parsed from the “_struct_conn” and “_pdbx_entity_branch_link” categories as edges. If the ligand is composed of multiple CCDs linked by covalent bonds, then the graph object of the ligand is converted to a Component object in PDBe CCDUtils by parsing information about atoms and 3D coordinates from “_atom_site”, adding bonds within each CCD from “_chem_comp_bond” and adding bonds among CCDs from edges between nodes of the graph object described above. Finally, hydrogen atoms are computationally added using RDKit to create the Component object of the multicomponent ligand. If the component's InChIKey does not match any existing reference small molecules (CCDs, PRDs, or previously identified CLCs), it is classified as a new Covalently Linked Component (CLC). As part of the weekly PDBeChem pipeline at PDBe [26], this new CLC is assigned a unique identifier, facilitating identification and access to these molecules.

### 2D image generation of ligands in PDB

RDKit offers various methods for generating 2D depictions of ligands [30], but no single method is universally optimal for all the ligands in the PDB. Hence, we defined a score to quantify the quality of 2D depictions and select the best image generated by the template-based or connectivity-based methods of PDBe CCDUtils. The template-based method generates 2D depictions using a template molecule via the RDKit function “rdkit.Chem.AllChem.generateDepictionMatching2DStructure”. The template can be user-provided or downloaded from PubChem [31]. A hand-curated set of ten templates are also provided with PDBe CCDUtils which is available at [32]. If a user chooses to use a template from PubChem, PDBe CCDUtils uses the PubChem API [33] to download the template molecule based on the InChIKey match. The PubChem templates are rescaled to a bond length of 1.5 Å before generating depictions, as it is the default bond length of depictions generated by RDKit. The connectivity-based method uses the “rdkit.Chem.rdCoordGen” module to generate 2D coordinates.

PDBe CCDUtils runs both methods for all ligands, and a Depiction Penalty Score is calculated to determine the best image. We observed that the quality of the 2D depiction deteriorates when bonds collide and atoms crowd together in the 2D space. To account for this, we defined the Depiction Penalty Score (DPS) as a weighted sum of the number of bonds colliding and the number of pairs of atoms in suboptimal positions:

$${\text{DPS }} = \, \alpha {\text{ n}}_{\text{b}} + \, \beta {\text{ n}}_{\text{p}}$$

where α is the bond collision penalty, n_b_ is the number of bonds colliding, β is the suboptimal atom position penalty, and n_p_ is the number of pairs of atoms closer to each other than the RDKit’s standard distance. We set α to 1 and β to 0.4 by default. A DPS of zero indicates a high-quality 2D depiction, while higher scores indicate lower quality. To count the number of bonds colliding, we considered two cases. First, when an atom is shared between two bonds, we consider the bonds to be colliding if the angle between them is less than 10°. Second, when no atoms are shared between two bonds, Cramer's rule is used to check for collisions [34]. Similarly, we classified pairs of atoms to be in suboptimal positions if the distance between them is less than 0.5 Å. To consider such pairs of atoms in suboptimal positions, we counted the number of atoms within 0.5 Å radius of each atom using the “spatial.KDTree.query_ball_point” function from SciPy [35].

### Scaffolds and a curated library of fragments

Scaffolds are core chemical substructures characterising a group of molecules [36]. Their relevance is reflected in those compounds sharing the same scaffold are likely to have similar synthetic pathways [36]. Consequently, scaffolds with preferable biological activities are often used as initial templates for compound synthesis and diversification in small molecule drug discovery [37]. Although there are various definitions for molecular scaffolds, PDBe CCDUtils uses the most commonly used methods implemented in RDKit—MurckoScaffolds [38] and BRICS [39]. These algorithms can be easily accessed to generate scaffolds of small molecules in PDB via the ‘get_scaffolds’ method of the Component object. Fragments are small chemical structures that may contribute to binding to a target macromolecule [40]. In fragment-based drug discovery, the potency of small fragments with relatively weak binding affinities can be improved by chemically combining them to form larger structures with higher specificity and improved binding characteristics [40]. PDB ligands can be searched against a library of 2158 fragments manually curated by PDBe [26], ENAMINE [41] and Diamond-SGC-iNext Poised Library (DSiP) [42] using the ‘library_search’ method. Alternatively, an external library of fragments can also be supplied as a tab-delimited file in the same format as the default fragment library in PDBe CCDUtils, which is available at [43].

## Results and discussion

The PDBe CCDUtils package provides a range of functionalities for analysing and manipulating small molecules in PDB structures. Here we provide examples of how PDBe CCDUtils can be used in a scientific context.

### Identification of CLCs in PDB entries

**Fig. 3:**
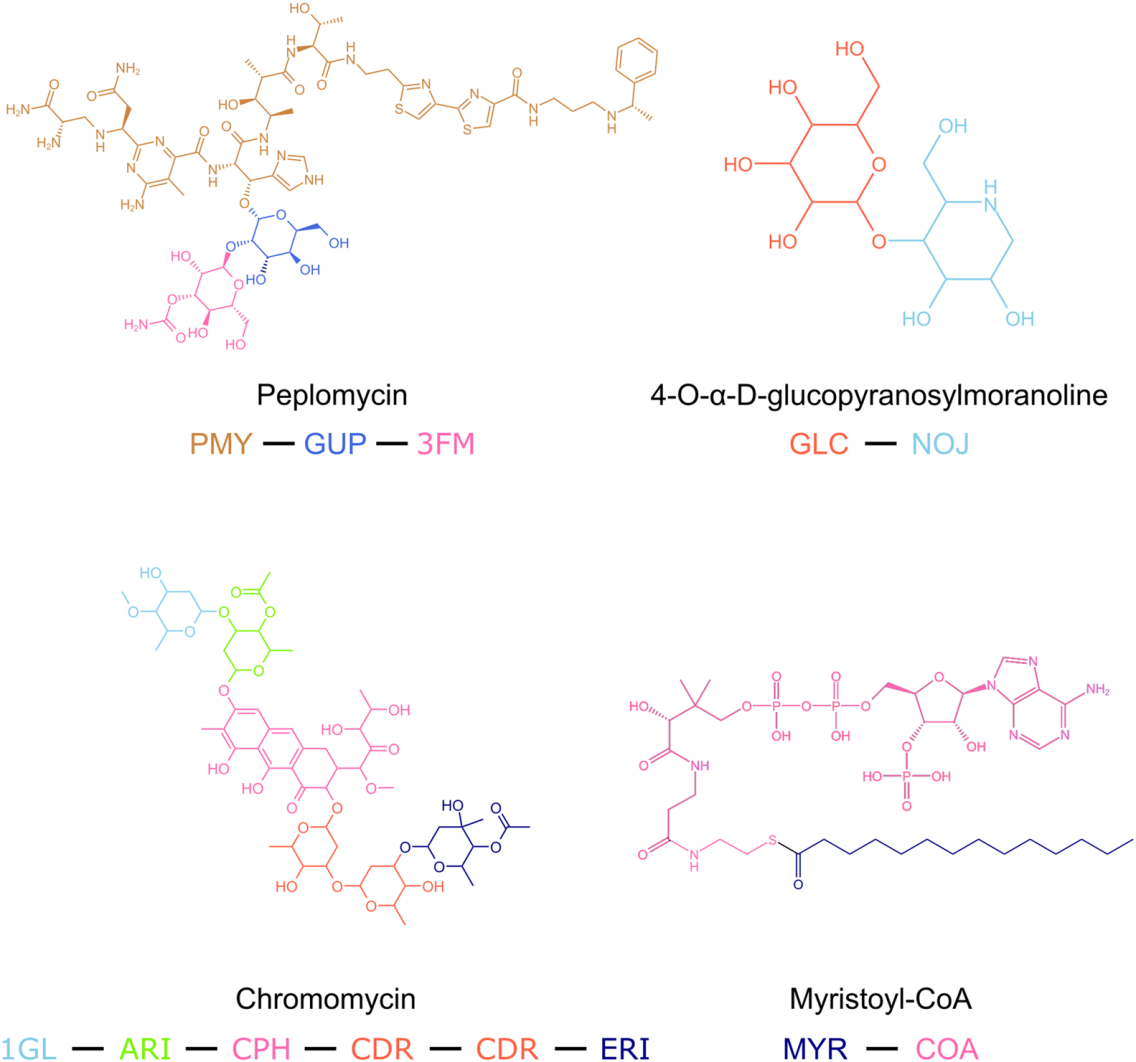
Examples of CLCs. Examples of Covalently Linked Components (CLCs) coloured based on their constituting chemical components (CCDs)

Figure 3 showcases four examples of Covalently Linked Components (CLCs) identified using PDBe CCDUtils in different PDB entries. These representations highlight the complete chemical structure of CLCs, which were previously fragmented into multiple CCD components during PDB deposition. Leveraging the InChIKeys of these CLCs, we were able to map and cross-reference these molecules to various external databases such as DrugBank, ChEMBL, ChEBI, and PubChem as shown in Table 1 and is available to the user in CIF files available at PDBe FTP area [24]. Such cross-references to other external databases facilitate easier access to valuable information about the biological and chemical contexts in which these molecules are found, providing researchers with deeper insights into their functional and pharmacological relevance.

**Table 1:** Examples of CLCs with cross-references to other external resources

Name	CLC Identifier	Representative PDB Entry	External Database Cross-references
Chromomycin A3	CLC_000153	1d83	CHEMBL2303619 PubChem: 656673 ChEBI: 34638
Peplomycin	CLC_000034	1ao4	CHEMBL3990196 PubChem: 6852373 ChEBI: 135909
Myristoyl-CoA	CLC_002763	6lq4	DrugBank: DB02180 PubChem: 11966124 ChEBI: 15532
4-O-ɑ-d-glucopyranosylmoranoline	CLC_001242	2xg9	PubChem: 11324809 ChEBI: 70736 BRENDA: 167734

### Indication of quality of 2D images using the DPS

**Fig. 4:**
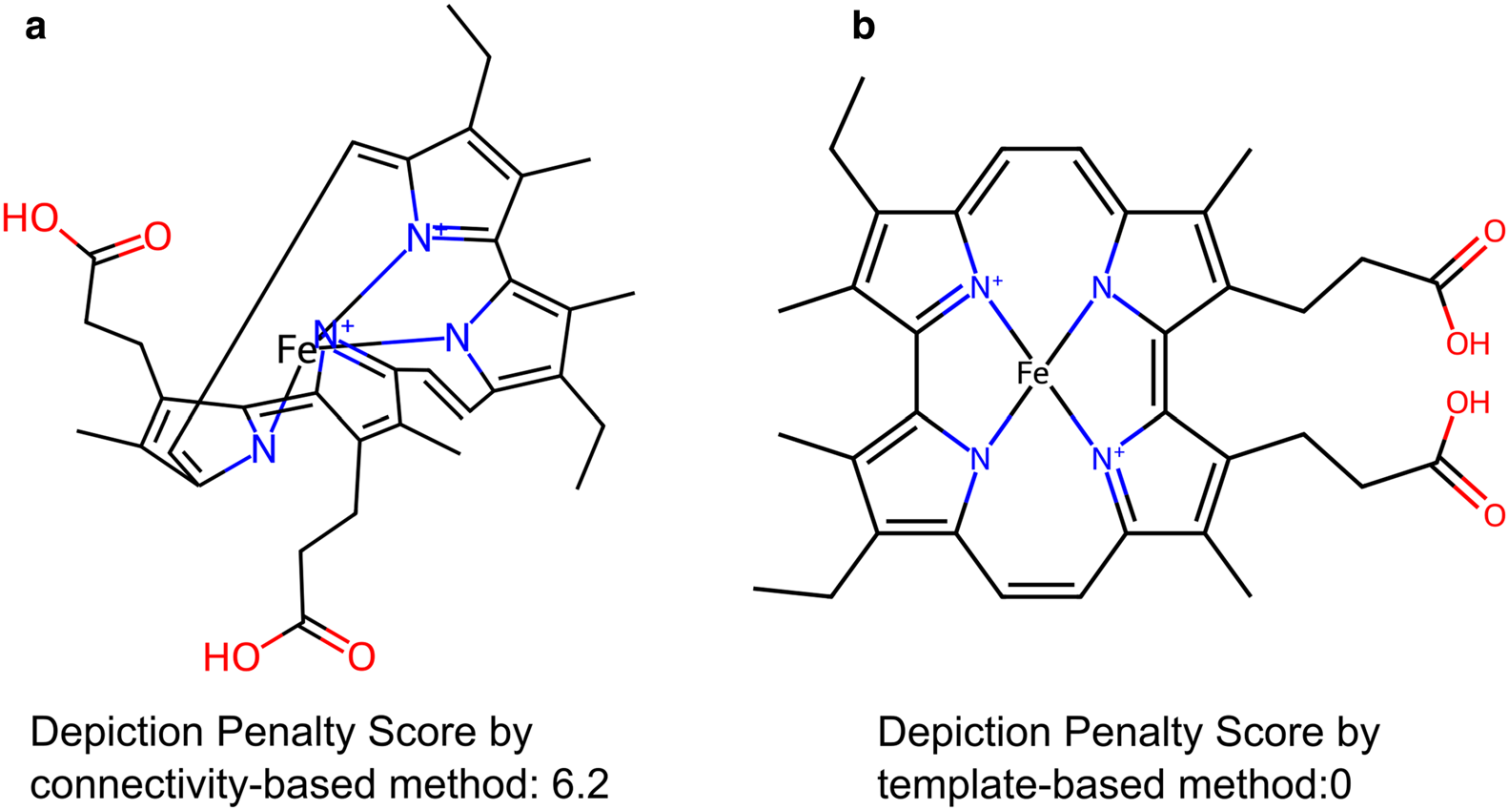
Example for use of Depiction Penalty Score. The Depiction Penalty Score is a metric to assess the quality of 2D depictions generated using RDKit. **a** 2D depiction of ‘HME’ with a high Depiction Penalty Score, highlighting some minor bond clashes and suboptimal atom distances in the 2D depiction, generated using the connectivity-based method. **b** 2D depiction of ‘HME’ with a Depiction Penalty Score of 0, indicating a high-quality representation with no bond clashes generated using the template-based method

The DPS is a valuable metric to assess the quality of 2D depictions generated using RDKit [44]. A DPS of zero indicates that there are no bond clashes, and there is adequate distance between atoms in the 2D depiction, resulting in high-quality 2D representation. As the value of DPS increases, the quality of the 2D depiction deteriorates. Figure 4 presents an example of 2D depictions generated for the CCD component ‘HME’ (porphycene containing iron) using the connectivity-based and the template-based methods in PDBe CCDUtils. The template-based method generates a high-quality 2D depiction without bond-clashes using the Porphycene template in PDBe CCDUtils. The best 2D image and 2D coordinates for each CCD, PRD and CLC based on the DPS is available from the PDBe FTP area [24].

### Identification of scaffolds and fragments

**Fig. 5:**
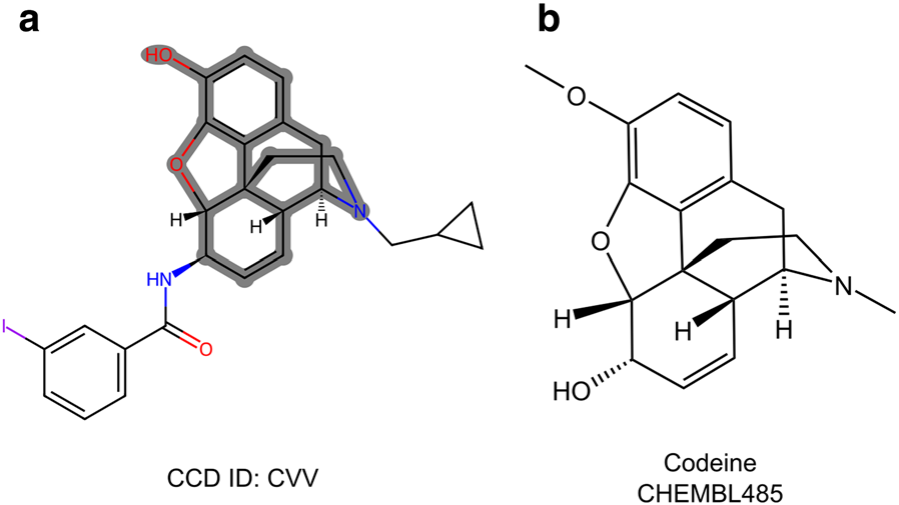
Example for identification of scaffolds. **a** 2D depiction of CCD component CVV highlighted with scaffold identified by PDBe CCDUtils. **b** 2D depiction of Codeine

Medicinal chemists widely use the concept of classifying compounds based on their molecular scaffolds to group molecules with similar properties [45]. Figure 5a showcases the scaffold identified by PDBe CCDUtils using the BRICS fragmentation rule [39] for the CCD component CVV when bound to the human kappa opioid receptor (PDB entry 6b73). Interestingly, it is an exact match to the scaffold of Codeine (ChEMBL485), depicted in Fig. 5b. Codeine is a known analgesic that targets various opioid receptors [46], and its biological activity is well-documented in ChEMBL [47]. Although the PDB does not contain the structure of Codeine, the shared scaffold between Codeine and CCD component CVV suggests that Codeine may interact with the Human kappa opioid receptor in a similar manner to CVV.

Furthermore, the PDBe CCDUtils' fragment library was used to identify matching fragments to CVV, and these are listed in Table 2. This process allows researchers to explore smaller molecular fragments that can potentially interact with the target of interest and serve as starting points for drug development [48]. By understanding the shared scaffolds and fragments, medicinal chemists can make informed decisions and design new compounds with improved pharmacological properties and higher chances of success in drug discovery [49].

**Table 2:** Matching fragments of CVV from PDBe CCDUtils’ fragment library

Name	SMILES	Structure	Fragment library source
Amide	CC(N) = O		PDBe
Benzofuran	c1ccc2c(c1)CCO2		PDBe
Cyclopropane	C1CC1		PDBe
Phenyl	c1ccccc1		PDBe
Z419084966	NCC(O)Cc1ccccc1		ENAMINE

### Additional small molecule related meta-data

PDBe CCDUtils serves as the primary package in the PDBeChem pipeline, a weekly update process for the small molecule information in PDB [26]. The pipeline processes three key PDBx/mmCIF reference files related to small molecules: CCDs, PRDs and CLCs, enriching them with valuable additional information. This additional information encompasses RDKit-generated 3D conformers, 2D coordinates of the ligands, RDKit-generated physicochemical properties and details about scaffolds and fragments. Moreover, the pipeline also establishes cross-references to other external small molecule databases like ChEMBL, PubChem, KEGG and DrugBank through UniChem cross-references [50]. Additional information on descriptions, synonyms, taxonomy, and known targets are added for the small molecules mapped to DrugBank. Synonyms collected from ChEMBL and wwwPDB are also added to the updated PDBx/mmCIF files. All the files generated by the PDBeChem pipeline are available from the PDBe FTP area [24]. This directory contains a detailed readme file elucidating the contents, along with separate folders for each enriched small molecule reference file: CCD, PRD, and CLC. Each CCD/PRD or CLC identifier has this data in various types of files, further elaborated in Table 3.

**Table 3:** Details of enrichments and associated files for CCD, PRD or CLC identifiers generated by the PDBeChem pipeline using PDBe CCDUtils

File suffix	Description	Available for
.cif	Standard wwPDB CCD file with enriched data: UniChem cross-references, RDKit-generated conformers, physicochemical properties, 2D coordinates, scaffolds, fragments	CLC, PRD, CCD
_ideal.pdb	Ideal coordinates in PDB format	PRD, CCD
_ideal_alt.pdb	Ideal coordinates with alternate names of atom	PRD, CCD
_model.pdb	Model coordinates	CLC, PRD, CCD
_model_alt.pdb	Model coordinates with alternate names of atoms	PRD, CCD
_N.svg	2D depictions in *N* x *N* pixels. Where *N* is 100, 200, 300, 400, or 500	CLC, PRD, CCD
_N_names.svg	2D depictions in *N* x *N* pixels with atom names. Where N is 100, 200, 300, 400, or 500	CLC, PRD, CCD
_model.sdf	Model coordinates in SDF format	CLC, PRD, CCD
_ideal.sdf	Ideal coordinates in SDF format	PRD, CCD
.cml	Model coordinates in CML format	CLC, PRD, CCD
_annotation.json	2D depiction in JSON format with annotations	CLC, PRD, CCD

## Future work

While PDBe CCDUtils expands the functionality of RDKit, it does come with certain limitations. PDBe CCDUtils presently only supports the parsing and processing of small molecule reference files (CCDs/PRDs) in mmCIF format. We plan to extend the functionality of PDBe CCDUtils to process small molecule data directly from the PDB entry file. Additionally, a distinct package is in active development, specifically designed for analysing the interactions of small molecules leveraging PDBe CCDUtils for more accurate ligand definitions. Another limitation lies in the current data sanitisation process implemented in PDBe CCDUtils. It primarily addresses valence concerns specific to metal atoms as identified by RDKit. As of 18 October 2023, approximately 0.5% (188 out of 41,400) of chemical components encountered difficulties with the sanitisation procedure in PDBe CCDUtils. Notably, recent updates in RDKit (2023.03, 2023.09 version) have significantly improved its support for organometallic compounds with the inclusion of a cleanup step designed to convert single bonds between metal atoms and hypervalent atoms into more precise coordinate bonds. These RDKit enhancements positively impact the handling of metal-containing compounds within PDBe CCDUtils and some of the encountered issues may find resolution with the adoption of this updated RDKit version. To further enhance our quality control of 2D depictions, the integration of AI-based tools like Deep Learning for Chemical Image Recognition (DECIMER) [51] can be explored.

## Conclusions

PDBe CCDUtils is a versatile Python package designed to streamline the analysis and manipulation of small molecules in the Protein Data Bank (PDB) by expanding the core functionalities of RDKit. This tool addresses the challenges faced by researchers working with a diverse range of small molecules in the PDB, offering an accessible and efficient toolkit tailored to meet the needs of researchers in cheminformatics, structural biology, bioinformatics, and computational chemistry.

The integration of RDKit with PDBe CCDUtils allows users to leverage the powerful features provided by RDKit while offering additional enhancements, such as the definition of CLCs for large, complex ligands, which are often split into individual CCDs in the PDB. This approach enables a more accurate representation and analysis of complex ligands in biological systems, with the added functionality of generating unique InChIKeys for each CLC molecule.

## Data Availability

The data used for the analysis are available from PDBeChem FTP: https://ftp.ebi.ac.uk/pub/databases/msd/pdbechem_v2/. *Availability and requirements* Project name: ccdutils. Project home page: https://github.com/PDBeurope/ccdutils. Operating systems: Platform independent. Programming language: Python. Other requirements: Python 3.9+; RDKit 2022.09.4 or higher. Licence: Apache 2.0. Any restrictions to use by non-academics: see the licence. Tutorials: https://pdbeurope.github.io/ccdutils/guide/intro.html.
